# Langfristige Therapiekonzepte bei Osteoporose

**DOI:** 10.1007/s00108-021-00993-3

**Published:** 2021-03-12

**Authors:** Barbara Obermayer-Pietsch, Ines Fössl, Hans Peter Dimai

**Affiliations:** Klin. Abteilung Endokrinologie und Diabetologie, Univ. Klinik für Innere Medizin, Medizinische Universität Graz, Auenbruggerplatz 15, 8036 Graz, Österreich

**Keywords:** Pharmazeutika, Medikamentenbezogene Begleiterscheinungen und Nebenwirkungen, Compliance, Patienten-Monitoring, Forschung und Entwicklung, Pharmaceuticals, Drug-related side effects and adverse reactions, Compliance, Patient monitoring, Research and development

## Abstract

Die Notwendigkeit einer Langzeittherapie bei Osteoporose, eine teils eingeschränkte Compliance, aber auch die Möglichkeit von erheblichen Nebenwirkungen bei einer pharmakologischen Osteoporosetherapie beschäftigen sowohl die medizinischen Richtlinien als auch die Betroffenen in vielfacher Weise. In dieser Übersicht wird auf den Stand der zur Verfügung stehenden Osteoporosepharmazeutika und die aktuellen wissenschaftlich fundierten Grundlagen einer langjährigen Anwendung, das potenzielle Monitoring und mögliche Therapieänderungen mit dem spezifischen Augenmerk auf künftige Entwicklungen eingegangen.

## Abstract (en)

The need for a long-term pharmacological treatment of osteoporosis, the problem of potential compliance issues and also potentially severe side effects during the treatment are of central interest not only for patients but also for medical guidelines and prescribers. This review summarizes the current knowledge about the pharmacological substances used and the current scientifically based guidelines and approaches for the long-term use as well as the monitoring and potential treatment changes with a special focus on future developments.

Für die aktuell zugelassenen Osteoporosetherapeutika – hier geht es um p.o.- und i.v.-Bisphosphonate, Denosumab, Teriparatid, Raloxifen/Bazedoxifen und Romosozumab – sind positive Effekte gegen den Verlust an Knochenmasse und für eine Reduktion von Knochenbrüchen bei Frauen und Männern in unterschiedlichem Ausmaß nachgewiesen worden. Bekannt sind neben akuten Medikamentenreaktionen aber auch Langzeitnebenwirkungen, deren Management durchaus komplex sein kann. Die Furcht vor solchen Komplikationen kann Osteoporosebetroffene sogar davon abhalten, die verschriebene Medikation – noch dazu auf jahrelange Sicht – anzuwenden. Wie lang therapiert werden soll, ob und wann es Therapiepausen gibt, und welche neuen Konzepte zum Monitoring von Osteoporosetherapien es gibt, wird in den einzelnen Abschnitten dieser Übersicht erörtert.

## Pharmakologische Therapie der Osteoporose

Die aktuell zur Verfügung stehenden Osteoporosepharmazeutika lassen sich entsprechend ihrem vorwiegenden Wirkmechanismus als antiresorptiv, osteoanabol oder dual wirksam klassifizieren. Die vorwiegend *antiresorptiv* wirksamen Pharmazeutika umfassen selektive Östrogenrezeptormodulatoren (SERM) wie etwa Raloxifen oder Bazedoxifen, Bisphosphonate sowie das Biologikum Denosumab. Das einzige derzeit in der Europäischen Union zugelassene vorwiegend *osteoanabol* wirksame Pharmazeutikum ist Teriparatid (rekombinantes humanes, biologisch aktives N‑terminales Fragment [rhPTH 1‑34] des humanen Parathormons). In der Substanzklasse der *dual* wirksamen Pharmazeutika ist Romosozumab alleiniger Vertreter, zumal Strontiumranelat im Jahr 2017 vom Markt genommen wurde [1, 2]. *Raloxifen und Bazedoxifen* sind nicht nur für die Behandlung, sondern auch für die Prävention der postmenopausalen Osteoporose zugelassen. Der relativ moderate Effekt auf die Knochenmineraldichte (KMD) ist mit einer deutlichen Reduktion des vertebralen Frakturrisikos verbunden.

Eine *Hormontherapie* ist bei postmenopausalen Frauen mithilfe der Östrogen-Gestagen-Therapie oder (bei hysterektomierten Frauen) mithilfe der Östrogenmonotherapie prinzipiell unter entsprechenden Indikationen möglich und erzielt sowohl Anstiege der KMD als auch eine Reduktion vertebraler, Hüft- bzw. nonvertebraler Frakturen [3]. Dies gilt ebenso für eine Androgenersatztherapie bei Männern. Aufgrund der Diversität dieser Substanzgruppen, der fehlenden Zulassung zur Behandlung der (postmenopausalen) Osteoporose und des aktuellen Fokus des vorliegenden Beitrags auf eine Osteoporosemedikation im engeren Sinne wird hier auf adäquate Literatur verwiesen [4].

Für alle *Bisphosphonate* ist bei unterschiedlichem Effekt auf die KMD eine Reduktion des vertebralen Frakturrisikos gesichert, wobei nicht für alle zugelassenen Bisphosphonate und deren jeweils unterschiedliche Galenik Ergebnisse aus aussagekräftigen randomisierten, kontrollierten Studien vorliegen. So liegt beispielsweise für die i.v.-Galenik von Ibandronat, das in 3‑monatlichen Intervallen appliziert wird, keine randomisierte, kontrollierte Studie mit dem primären Endpunkt des Frakturrisikos vor, sehr wohl aber für die einmal monatlich p.o. zu verabreichende Galenik. Das derzeit potenteste zur Behandlung der postmenopausalen Osteoporose zugelassene Bisphosphonat ist Zoledronat. Positive Effekte sind nicht nur hinsichtlich vertebraler und nonvertebraler Frakturrisikoreduktion gesichert, sondern auch hinsichtlich einer reduzierten Mortalität bei Personen mit vorangegangener Hüftfraktur. Auch ist es derzeit das einzige Bisphosphonat, für das eine Reduktion des vertebralen Frakturrisikos bei Männern belegt ist [5].

Für *Denosumab*, das erste in der Behandlung der postmenopausalen Osteoporose zugelassene Biologikum, ist ebenfalls ein positiver Effekt auf das vertebrale und nonvertebrale Frakturrisiko belegt. Im Unterschied zu den Bisphosphonaten wird Denosumab jedoch nicht renal eliminiert, weswegen eine eingeschränkte Nierenfunktion per se keine Kontraindikation darstellt. Dementsprechend konnte gezeigt werden, dass der positive Effekt auf das Frakturrisiko auch bei eingeschränkter Nierenfunktion (einschließlich Stadium IV einer „chronic kidney disease“, CKD) erhalten bleibt [5].

Adäquate *Kalzium- und Vitamin-D‑Gaben* sollten in Abhängigkeit von der spezifischen Situation jedenfalls als Basistherapie durchgeführt werden.

## Guidelines zur osteoporosespezifischen Medikation

### Aktuelle Empfehlungen zur Dauer der Osteoporosemedikation

In der aktuell gültigen Fassung der Leitlinien [6], erarbeitet durch den Dachverband Osteologie (DVO), soll eine spezifische Osteoporosetherapie nach jeweils 3 bis 5 Jahren Therapiedauer hinsichtlich Nutzen und Risiko reevaluiert werden (Empfehlungsgrad A, Evidenzgrad 2+, Konsensstärke: starker Konsens). Hierbei sind die persönliche Situation und evtl. Zusatzerkrankungen oder eine geänderte Lebenssituation der Betroffenen zu berücksichtigen.

Im Besonderen wurden für einzelne Osteoporosetherapeutika folgende eigenen Zeiträume definiert:

- Die Therapie mit Teriparatid ist generell auf 24 Monate begrenzt.
- Für eine Therapie mit Raloxifen besteht ein nachgewiesener Nutzen bis zu 8 Jahren.
- Die Therapie mit Bisphosphonaten hat einen nachgewiesenen Nutzen für 3 bis 5 Jahre.
- Denosumab hat einen nachgewiesenen Nutzen bis zu 3 Jahren. Nach dem Absetzen müssen aber andere Maßnahmen zum Erhalt der Knochendichte eingesetzt werden.

In Zusammenschau mit weiteren internationalen Guidelines ergibt sich ein erweitertes Bild. Die Fortführung einer Osteoporosetherapie – abhängig von der verwendeten Medikation – könnte u. a. folgenden Überlegungen folgen [4]:

- Bei Hochrisikopersonen wurde die Fortführung einer Bisphosphonattherapie nach der empfohlenen Risikoevaluierung nach 3 bis 5 Jahren angedacht.
- Raloxifen bewirkt zusätzlich eine über mindestens 5 Jahre nach Behandlungsende hinausgehende Reduktion des Brustkrebsrisikos.
- Eine Einschätzung des Frakturrisikos bei Personen mit Denosumabtherapie sollte nach einer Therapiedauer von 5 bis 10 Jahren nochmals durchgeführt werden. Absetzen oder Pausieren des Medikaments sollte aktuell nur unter Wechsel auf alternative Antiresorptiva erfolgen, da es zu einem Rebound-Phänomen kommen kann, das mit einem deutlich erhöhten Knochenumsatz und einem hohen Frakturrisiko behaftet ist [7].
- Zum Erhalt der Knochenmasse ist eine konsequente antiresorptive Therapie nach dem Ende einer 2‑jährigen Applikation von Teriparatid unerlässlich.

**Figure 1:**
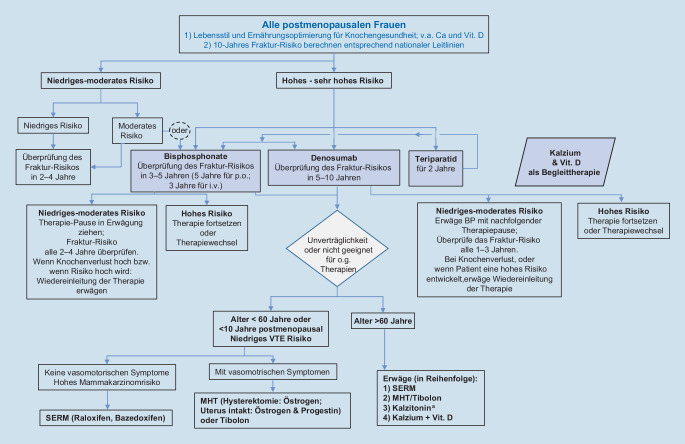

Diese und viele weitere Aspekte einer Langzeittherapie werden in den kommenden Abschnitten ausführlich diskutiert (Abb. 1).

Basierend auf einer Messung der KMD mithilfe der Zwei-Spektren-Röntgenabsorptiometrie („dual energy x‑ray absorptiometry“, DXA) an Hüfte und Wirbelsäule wurde eine Risikoabschätzung mithilfe des Fracture Risk Assessment Tool (FRAX, www.sheffield.ac.uk/FRAX) zugrunde gelegt.

### Probleme der Compliance und Adhärenz

Eine verminderte Compliance (i.e. Therapietreue) bei oralen Formen der Osteoporosetherapie nährt sich aus der primär notwendigen Langzeitbehandlung, aus akuten (gastrointestinalen) Nebenwirkungen, aber auch aus gefürchteten Langzeitnebenwirkungen (s. Abschn. „Langzeiteffekte osteoporosespezifisch wirksamer Pharmazeutika auf Knochenmineraldichte und Frakturrisiko“). Hinzu kommen unterschiedliche individuelle Faktoren, wie nichtausreichende Aufklärung oder mangelnder Zugang zu gesichertem medizinischem Wissen.

Offenes Ansprechen patientenseitiger Wünsche und Vorstellungen verbessert die Adhärenz

Eine geringere Adhärenz (i.e. Einhaltung der Therapieziele, eine Kombination aus Compliance und Persistenz) bei oralen Formen der Osteoporosetherapie ist bekannt. Sie wurde für Europa und Nordamerika mit 30 % im ersten Jahr [4] eingeschätzt und hat neben der fehlenden Frakturrisikoverminderung auch deutliche Folgen für die Kosteneffizienz von Osteoporosetherapien [8]. Daher ist es wichtig, patientenseitige Wünsche und Vorstellungen für eine verbesserte Adhärenz offen anzusprechen.

## Langzeiteffekte osteoporosespezifisch wirksamer Pharmazeutika auf Knochenmineraldichte und Frakturrisiko

Der überwiegende Teil randomisierter, kontrollierter Studienphasen zu den für die Behandlung der postmenopausalen Osteoporose zugelassenen Medikamenten erstreckt sich über einen Zeitraum von 36 Monaten. Behandlungszeiträume von mehr als 3 Jahren gelten daher i. Allg. als Langzeittherapie [9]. Effekte einer Langzeittherapie können unter dem Aspekt der KMD-Veränderungen und der (persistierenden) Frakturrisikoreduktion, aber auch der möglichen Nebenwirkungen (s. Abschn. „Mögliche Nebenwirkungen osteoporosespezifisch wirksamer Pharmazeutika“) betrachtet werden.

Untersuchungen zum Verlauf der KMD liegen für Alendronat und Denosumab über einen Behandlungszeitraum von maximal 10 Jahren vor, für Zoledronat über einen Zeitraum von 9 Jahren. Während der KMD-Verlauf an der LWS unter Zoledronat nach einem initial steileren Anstieg insbesondere in den Jahren 6 bis 9 bis zu einer Plateaubildung abflacht, ist unter Alendronat und Denosumab selbst im 10. Behandlungsjahr noch ein weiterer Anstieg zu erkennen.

Unter einer Langzeitbehandlung mit Denosumab ist die Reduktion des vertebralen und nonvertebralen Frakturrisikos vergleichbar mit jener innerhalb der ersten 3 Therapiejahre. Für eine relevante Beurteilung des Frakturrisikos unter Alendronat über einen Behandlungszeitraum von 10 Jahren bzw. Zoledronat über einen Behandlungszeitraum von 9 Jahren liegen keine ausreichend aussagekräftigen Daten vor.

Für Raloxifen und Bazedoxifen, ebenso für eine postmenopausale Hormonsupplementation, liegen keine vergleichbaren Daten vor.

## Mögliche Nebenwirkungen osteoporosespezifisch wirksamer Pharmazeutika

### Atypische Femurfrakturen

Eine seltene, aber sehr folgenreiche Nebenwirkung von Bisphosphonaten oder Denosumab kann eine atypische Femurfraktur (AFF) sein, die durch eine verminderte „Bruchzähigkeit“ am Femurschaft ohne oder bei nur geringen Traumata auftritt, wobei Subtypen von Knochenstoffwechselveränderungen eine Rolle spielen dürften [10]. Auch wenn das absolute Risiko einer AFF sehr niedrig im Vergleich zur therapiebedingten Verminderung von Hüftfrakturen ist, steigt diese Komplikation dennoch mit einer längeren Therapiedauer an, sinkt aber nach Absetzen der Therapie rasch ab [11]. Bei den anderen Substanzgruppen (Raloxifen, Teriparatid, Romosozumab) wurde diese Komplikation bisher nicht beobachtet; eine verbesserte Heilung von AFF unter Teriparatid wird diskutiert [12].

### Kieferosteonekrosen

In allen Klassen von Antiresorptiva – p.o.- und i.v.-Bisphosphonaten, aber auch Denosumab – sind Kieferosteonekrosen („osteonecrosis of the jaw“, ONJ) als schwere Komplikation nachgewiesen worden, die mit der Dauer der Therapie und der Frequenz/Höhe der Dosierung deutlicher werden. Eine Phaseneinteilung erstreckt sich von kaum vorhandenen Symptomen bis zu offen liegendem Kieferknochen [13]. Die Inzidenz beträgt bei einer Osteoporosestandardtherapie etwa 1:10.000 bis 1:100.000, bei onkologischen Indikationen mit kürzeren Therapieintervallen sind aber 1–9 % aller Behandelten in einem Ausmaß betroffen [14], das eine spezifische zahnmedizinische Versorgung erfordert. Für Osteoanabolika oder hormonelle Therapieformen sind keine derartigen Nebenwirkungen beschrieben. Da prädisponierende Faktoren (noch) weitgehend unklar sind – diskutiert werden genetische, aber auch mikrobielle und inflammatorische Risikofaktoren –, können vorbeugend nur eine Beschränkung der Applikationszeit mit entsprechenden Pausen (*Cave*: Denosumab) sowie zahnmedizinische und orale Hygiene vorgesehen werden.

### Vaskuläre Veränderungen und kardiovaskuläres Risiko

Aufgrund von gemeinsamen pathophysiologischen Mechanismen bei Osteoporose und kardiovaskulären Veränderungen sind einige Osteoporosetherapeutika mit einem erhöhten kardiovaskulären Risiko (Romosozumab, Kalziumsupplementation, Hormonersatztherapie), ohne spezielles Risiko (Vitamin D) oder sogar mit einem reduzierten Risiko (Bisphosphonate) verbunden. Im venösen System können Raloxifen und eine postmenopausale Hormonersatztherapie zu einem erhöhten Thromboembolierisiko führen. Eine Romosozumabtherapie ist bei Personen mit einer myokardialen oder zerebralen Ischämie in der Vorgeschichte kontraindiziert [15].

### Veränderungen in der Knochenumbildung und -heilung

Während eine Langzeittherapie mit Antiresorptiva (Bisphosphonate, Denosumab) histologische Veränderungen, u. a. auch der Angiogenese, und ein erhöhtes Risiko für die oben angegebenen Komplikationen mit sich bringen kann, sind Frakturheilungsstörungen im eigentlichen Sinne nicht beobachtet worden. Zu einigen Frakturen (wie etwa Wirbelkörperfrakturen) stehen für einzelne Therapeutika noch keine ausreichenden Daten zur Verfügung. Teriparatid wurde als positiv für den Knochenheilungsprozess und die dabei auftretende Schmerzsymptomatik beschrieben. Damit kann eine unmittelbare Therapieeinleitung nach Frakturen unterstützt werden.

## Auswirkungen einer Therapiepause/-beendigung

### Bisphosphonate

Bisphosphonate akkumulieren im Knochen und werden zeitabhängig nach der Therapiebeendigung aus diesem auch wieder freigesetzt. Die Wirkung von Bisphosphonaten kann daher auf diese Art Jahre nach deren Verabreichung noch anhalten, wenn auch in abnehmendem Ausmaß und abhängig von der Molekülstruktur. In der Verlängerungsphase der Zulassungsstudie von Alendronat erhielt eine Studiengruppe nach einer Behandlungsdauer von 5 Jahren über weitere 5 Jahre keine Therapie (mit Ausnahme von Kalzium und Vitamin D). In dieser Gruppe zeigte sich gegen Ende der 5 Jahre ein leichter Anstieg der alkalischen Phosphatase. Diese Beobachtung begründet die Sichtweise, dass periodische Therapiepausen – etwa 3 Jahre nach Beginn einer i.v.- und 5 Jahre nach Beginn einer p.o.-Bisphosphonat-Therapie – insbesondere bei Personen mit nicht sehr hohem Frakturrisiko, eine sinnvolle Möglichkeit der Therapieoptimierung und -ökonomisierung darstellen [16].

Die Wirkung von Bisphosphonaten kann Jahre nach ihrer Verabreichung noch anhalten

Die Auswirkungen einer Beendigung der Bisphosphonattherapie auf die KMD wurden in mehreren Studien untersucht, u. a. in der Verlängerungsgruppe der Zulassungsstudie von Alendronat in der täglichen p.o.-Dosierung [17]. Die Studiengruppe mit kontinuierlicher Therapie über 10 Jahre wies in den Jahren 5 bis 10 keine signifikante Änderung der KMD an der Hüfte sowie am distalen Unterarm auf, während bei der Studiengruppe mit 5‑jähriger Therapiepause am Ende ein signifikanten Verlust zu verzeichnen war. An der LWS war in der Zehnjahresbehandlungsgruppe eine kontinuierliche Zunahme der KMD zu beobachten, während in der Gruppe mit 5 Jahren Therapiepause die KMD de facto unverändert blieb.

In einer rezenten Arbeit wurde der Effekt einer 96-wöchigen Therapiepause im Anschluss an eine 2‑jährige Therapie mit Alendronat, Risedronat oder Ibandronat untersucht. Der KMD-Verlust der Hüftregion („Gesamt“) betrug am Ende der Therapiepause −1,6 %, derjenige an der LWS −0,6 %, ohne signifikanten Unterschied zwischen den genannten Bisphosphonaten [18].

### Denosumab

Eine Beendigung der bei Osteoporose halbjährlich zu verabreichenden Therapie mit Denosumab führt zur vollständigen und raschen Umkehr der knochenspezifischen Effekte. Der genannte Rebound-Effekt tritt allerdings nicht erst nach vieljähriger Behandlung auf, sondern bereits nach den beiden ersten verabreichten Injektionen. Etwa 6 Monate nach der letzten Injektion steigen die Knochenumsatzmarker, wenn auch individuell unterschiedlich, an. Nach durchschnittlich 9 Monaten werden die Ausgangswerte bei Therapiebeginn deutlich überschritten, und erst 30 Monate nach der letzten Injektion werden dieselben wieder erreicht [19].

Der Rebound-Effekt von Denosumab tritt bereits nach den beiden ersten verabreichten Injektionen auf

Die während der Behandlung erzielten KMD-Zuwächse, die an der Wirbelsäule nach 10 Jahren kontinuierlicher Behandlung durchschnittlich 22 % und an der Hüfte etwa 9 % betragen, sinken etwa 1 bis 2 Jahre nach Therapieende auf das Niveau zu Therapiebeginn. Mit dem Anstieg der Knochenumsatzmarker und dem Abfall der KMD einhergehend, steigt das Risiko für multiple vertebrale Frakturen, dies insbesondere bei Patientinnen, die bereits zu Therapiebeginn vertebrale Frakturen aufwiesen [20]. Das Ausmaß des Risikoanstiegs nimmt mit Dauer der Behandlung zu. Eine rezente Untersuchung weist darauf hin, dass nach Therapiebeendigung auch das Hüftfrakturrisiko steigen könnte [21].

### Teriparatid

In einer kleineren Beobachtungsstudie wurde der Effekt einer 24- (bis maximal 30-)monatigen Teriparatidtherapie ohne antiresorptive Nachbehandlung über 12 Monate beobachtet [22]. Ein Jahr nach Therapieende waren die Knochenumsatzmarker deutlich gesunken. Der KMD-Verlust betrug an der LWS bei Frauen rund 7 % und bei Männern rund 4 %. Die KMD an der Hüfte (Gesamtwert) hatte bei Frauen um etwa 4 % abgenommen, während sie bei Männern stabil blieb.

Hinweise auf den Frakturrisikoverlauf nach Therapieende können aus der Verlängerungsphase der 18-monatigen Zulassungsstudie abgeleitet werden. In diese waren sowohl Frauen inkludiert, die nach dem Ende der Teriparatidtherapie andere spezifische Osteoporosetherapeutika erhielten, als auch solche, denen außer Kalzium und Vitamin D keine weitere Therapie verabreicht wurde [22]. In letzterer Subgruppe war unter der zugelassenen Dosierung ein Jahr nach Therapieende das relative Frakturrisiko im Vergleich zur Placebogruppe um 37 % reduziert.

### Selektive Östrogenrezeptormodulatoren

Aussagekräftige Studien zu Auswirkungen einer Beendigung einer Raloxifentherapie im Hinblick auf biochemische Knochenumsatzmarker, KMD oder Frakturrisiko stehen derzeit nicht zur Verfügung.

## Monitoring und individuelle Entscheidungen

### Monitoring-Konzepte und „least significant change“

#### Labormarker

Knochenumsatzmarker geben im Monitoring einer pharmakologischen Medikation bei Osteoporose wichtige Hinweise, auch was Änderungen des Knochenstoffwechsels in Therapiepausen, nach Therapiewiederaufnahmen und insbesondere durch mangelnde Compliance bei diesen Langzeittherapien betrifft. Typischerweise werden *Resorptionsmarker* (Serum-CrossLaps [CTX] oder tartratresistente saure Phosphatase 5b [TRAP5b]) bei antiresorptiver Therapie (Bisphosphonate, Denosumab) deutlich vermindert; daher sind sie wichtige Marker auch für eine Adhärenzüberprüfung. *Formationsmarker* (alkalische Phosphatase [AP], im eigentlichen Sinne knochenspezifische AP [„bone alkaline phosphatase“, bAP], das Telopeptid des Typ-1-Prokollagens [P1NP], aber auch Osteocalcin) werden unter osteoanaboler Therapie erhöht. Sie dienen dem spezifischen Monitoring des Therapieeffekts, aber auch möglicher Reaktionen des Knochenstoffwechsels nach Absetzen der pharmakologischen Therapie oder bei eingeschränkter Compliance. Weitere wichtige Laborwerte im Monitoring einer Osteoporosetherapie sind Vitamin-D-Spiegel, aber auch Kalzium- und Phosphatwerte und Laborhinweise auf sekundäre Osteoporoseformen, wie in den DVO-Guidelines ausführlich dargestellt.

Nach Konzepten u. a. der Arbeitsgruppe um Eastell [23] können diese Labordaten in 2 kombinierten Ansätzen im Monitoring eingesetzt werden – „Wo liegt der aktuelle Wert?“

**Figure 2:**
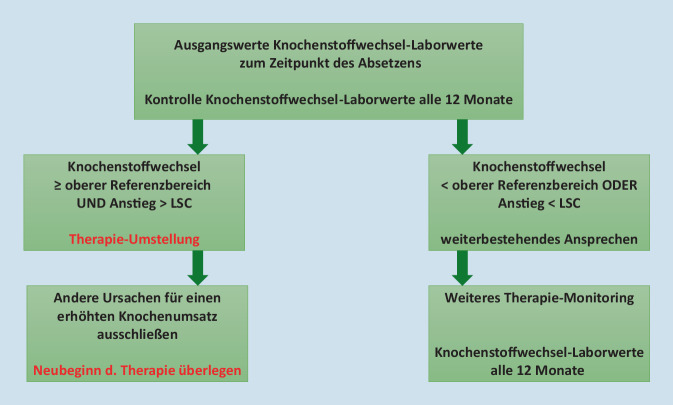

- orientiert an den Referenzbereichen junger gesunder Erwachsener und
- am individuellen LSC zwischen 2 Messzeitpunkten (Abb. 2 für das Bisphosphonat-Monitoring und Therapiepausen).

Ein LSC im Bereich der Knochenstoffwechselmarker wird als prozentuale individuelle Veränderung angegeben, um eine signifikante Änderung bei einem Individuum zwischen 2 Messzeitpunkten zu beschreiben. Je nach Knochenstoffwechselmarker sind erwartete Streuungen besonders bei Kollagenmarkern (CTX, P1NP) bis zu 50 % zu erwarten; bei Enzymen (bAP, TRAP5b) können es bis zu 20 % sein [24]. Daher ist die Erwartung für den Labor-LSC daran anzupassen.

#### Knochenmineraldichte

Für die Beurteilung der Frage, ob eine Änderung der KMD im Monitoring-Verlauf rein zufällig oder doch wahr ist, stellt der LSC ein unumgängliches Instrument dar. Er beinhaltet die institutionelle Präzision, die sich aus der Gerätepräzision sowie u. a. der Sorgfalt ergibt, mit der die zu messenden Personen im Monitoring-Verlauf repositioniert werden. Institutionelle Präzision und LSC können einfach mithilfe eines Online-Tools errechnet werden (iscd.org/learn/resources/calculators). Die Feststellung von Präzision und LSC wird als Qualitätsmerkmal seitens der International Society for Clinical Densitometry (ISCD) für jede Institution empfohlen. Für Verlaufskontrollen, deren Ergebnisse (in Gramm/Zentimeter zum Quadrat) sich außerhalb des institutionellen LSC befinden, kann mit 95 %iger Wahrscheinlichkeit angenommen werden, dass die Änderung wahr und nicht rein zufällig ist.

### Besondere Fälle

Aufgrund der potenziellen Retention einiger Osteoporosetherapeutika im Knochengewebe sind gerade junge Betroffene, insbesondere prämenopausale Frauen mit möglichem Kinderwunsch und Personen mit seltenen Knochenerkrankungen, außerhalb der bereits diskutierten Therapiekriterien zu sehen. Zur Therapieauswahl und zu individuellen Lösungen wird auf den diesbezüglichen Artikel dieses Schwerpunkthefts verwiesen (s. Beitrag von Seefried und Jakob).

### Personalisierte Therapie und klinische Umsetzung

In den letzten Jahren hat sich, auch unter dem Aspekt der genaueren Kenntnis pathophysiologischer Hintergründe für zahlreiche Erkrankungen ein neuer Zugang zu Indikation und Dosierung von Medikamenten eröffnet – eine „personalisierte Therapie“ soll diese individuelle Disposition der Patientinnen und Patienten berücksichtigen. Tatsächlich werden trotz aktuell zur Verfügung stehender Osteoporosetherapeutika noch immer zu wenige Patientinnen und Patienten therapiert bzw. nutzen diese Therapieoption nicht – großteils aus Angst vor Nebenwirkungen. Diese Ängste sollten wahrgenommen und über eine patientengerechte Aufklärung zu kurz-, mittel- und langfristigen Nebenwirkungen auch adressiert werden. Dabei können Monitoring-Untersuchungen wie Knochenstoffwechselmarker, DXA-Messungen, aber auch das Registrieren von Prodromalsymptomen dazu beitragen, den Patientinnen und Patienten mehr Sicherheit bei dieser A‑priori-Langzeittherapie zu geben.

Hochrisikopersonen für pharmakologische Nebenwirkungen zu identifizieren, u. a. über pharmakogenetische Risikomarker, ist Gegenstand der aktuellen Forschung und wird den Zugang zur personalisierten Osteoporosetherapie in Zukunft verbessern helfen.

## Zukünftige Entwicklungen – Kombinationstherapie und neue Therapieformen

Die zur Behandlung der Osteoporose zugelassenen Pharmazeutika werden in der Regel als Monotherapie verabreicht. Zukünftige Entwicklungen könnten neue Perspektiven eröffnen.

### Kombinierte Therapie

Eine kombinierte Therapie setzt voraus, dass zumindest 2 der zur Verfügung stehenden spezifischen Osteoporosetherapeutika parallel verabreicht werden. Kalzium und Vitamin D gelten nicht als Teile einer Kombinationstherapie. Die wenigen zur Verfügung stehenden Studien basieren insgesamt auf eher geringen Fallzahlen [1]. So zeigt die kombinierte Anwendung von 2 potenten Antiresorptiva im Hinblick auf eine Zunahme der KMD keinen Vorteil gegenüber den Einzelsubstanzen. Eine Kombination von Teriparatid mit Alendronat scheint zu einer Verringerung des anabolen Effekts von Teriparatid zu führen. Die zusätzliche Verabreichung von Teriparatid bei mit Alendronat vorbehandelten Personen bewirkt allerdings einen stärkeren Anstieg der KMD im Vergleich zu einer Fortsetzung der alleinigen Alendronattherapie. Die Kombination einer einmal jährlichen i.v.-Gabe von 5 mg Zoledronat mit Teriparatid in der Standarddosierung resultiert nach 52 Wochen in einer Zunahme der KMD an der LWS, vergleichbar einer solchen unter Teriparatid allein. Die KMD-Zunahme an der Hüfte fällt für die Kombinationstherapie vergleichbar mit jener unter Zoledronat allein aus, und beide Therapieformen sind einer Teriparatidmonotherapie an dieser Skelettregion überlegen [25].

### Sequenzielle Therapie

Für den klinischen Alltag von größerer Bedeutung sind sequenzielle Therapiemöglichkeiten, zumal sich solche vielfach nicht vermeiden lassen [1]. Unverträglichkeit und Nichtansprechen auf bestimmte Therapieformen zählen zu den häufigsten Ursachen. Ähnlich wie bei den kombinierten Therapieformen sind auch hier – mit einer einzigen Ausnahme (VERO-Studie [26–28]) – die Ergebnisse aufgrund der jeweiligen Fallzahlen und des Studiendesigns mit Vorsicht zu interpretieren.

*Antiresorptiv auf antiresorptiv*: Ein Wechsel von Alendronat auf Denosumab führt im Verlauf zu einer deutlicheren Zunahme der KMD an der LWS und der Hüfte im Vergleich zu einer Fortsetzung der Alendronattherapie. Ein Wechsel von Alendronat zu Denosumab oder Zoledronat bewirkt eine ausgeprägtere Zunahme der KMD an der LWS und Hüfte unter Denosumab.

*Osteoanabol oder dual auf antiresorptiv*: Generell wird der knochenanabole Effekt von Teriparatid durch eine vorangegangene antiresorptive Therapie eher abgeschwächt. Dennoch ist die Frakturrisikoreduktion bei Personen, die nach mehrjähriger Risedronattherapie Teriparatid erhielten, vergleichbar mit jener bei unbehandelten Patienten (VERO-Studie).

Teriparatid führt bei Personen, die zuvor über 2 Jahre mit Denosumab behandelt wurden, an der Hüfte zu einer signifikanten Abnahme der KMD. An der LWS steigt die KMD jedoch nach einer initialen Abnahme an (DATA-Switch-Studie). Die Kombination von Teriparatid und Denosumab scheint diesen Nachteil allerdings aufzuheben, auch bei Personen, die zuvor mit anderen Antiresorptiva behandelt wurden (DATA-Studie).

Bei Patientinnen und Patienten, die antiresorptiv vorbehandelt wurden, führt eine Behandlung mit Teriparatid ebenso wie eine Behandlung mit Romosozumab zu einer abgeschwächten KMD-Zunahme im Vergleich zu unbehandelten Personen (STRUCTURE-Studie [29]).

Bislang vorliegende Studienergebnisse zur sequenziellen Therapie sind mit Vorsicht zu interpretieren

Insgesamt erscheint der Effekt von osteoanabolen Substanzen auf die KMD ausgeprägter, wenn diese bei zuvor unbehandelten Personen verabreicht werden. Ein additiver Effekt einer osteoanabolen Therapie bei zuvor antiresorptiv (Denosumab ausgenommen) behandelten Personen ist dennoch sehr wahrscheinlich.

### Antiresorptiv auf osteoanabol oder dual

Die ossären Effekte osteoanabol oder dual wirkender Therapeutika sind grundsätzlich reversibel. Dies begründet die Überlegung, dass mit Abschluss einer osteoanabolen oder dual wirksamen Therapie eine „konsolidierende“ Anschlussbehandlung eingeleitet werden sollte. Eine Therapie mit Bisphosphonaten im Anschluss an eine Teriparatidbehandlung führt nicht nur zu einer weiteren Zunahme der KMD, sondern konsolidiert auch das unter Teriparatid gesunkene Frakturrisiko.

In einer komplex strukturierten Studie (CONFORS) wurde – bei zumindest über 2 Jahre mit einem Antiresorptivum vorbehandelten postmenopausalen Frauen – der Effekt einer 9‑monatigen Teriparatidbehandlung untersucht, die über einen Zeitraum von weiteren 9 Monaten um eine der 3 folgenden Anschlussbehandlungen ergänzt wurde: Teriparatidmonotherapie, Teriparatid kombiniert mit Raloxifen und Teriparatid kombiniert mit Alendronat. Danach wurde die Teriparatidgabe in allen 3 Gruppen beendet, und nach einem weiteren Jahr wurden die Therapieeffekte evaluiert [30]. Die KMD der LWS nahm in den beiden Kombinationstherapiegruppen deutlicher zu als in der Teriparatidmonotherapiegruppe. An der Hüfte fiel der KMD-Zuwachs am deutlichsten in der Teriparatid-Alendronat-Kombinationstherapie-Gruppe aus.

Die zukünftige Wahl von Kombinations‑/Sequenztherapien hängt auch von den Finanzierungsmodellen ab

Eine Anschlussbehandlung mit Denosumab führt nach einer 2‑jährigen Therapie mit Teriparatid zu einem weiteren Zuwachs der KMD an der LWS sowie der Hüfte (DATA-Switch Studie). Der Effekt einer Anschlussbehandlung mit Denosumab wurde auch nach einjähriger Vorbehandlung mit Romosozumab oder Placebo untersucht (FRAME-Studie). Nach 2 Jahren lag die KMD in der mit Romosozumab vorbehandelten Gruppe deutlich über jener mit Placebovorbehandlung. Die vertebrale Frakturrisikoreduktion war am Ende des zweiten Jahres vergleichbar mit jener am Ende des ersten Jahres.

Eine einmal wöchentliche Therapie mit Alendronat konsolidiert die unter einjähriger Vorbehandlung mit Romosozumab gewonnene KMD sowie das unter Romosozumabbehandlung gesenkte vertebrale Frakturrisiko (ARCH-Studie).

Welche Kombinations‑/Sequenztherapien in Zukunft angewandt werden, hängt neben möglicherweise individuell potenzierten oder auch vermindert auftretenden Nebenwirkungen u. a. von künftigen Finanzierungsmodellen ab [31]; die praktische Umsetzung ist daher abzuwarten.

### Neue Therapieformen

Romosozumab, ein humanisierter monoklonaler Sklerostinantikörper, wurde kürzlich sowohl vonseiten der Food and Drug Administration (FDA) als auch der European Medicines Agency (EMA) zur Behandlung der manifesten Osteoporose bei postmenopausalen Frauen mit deutlich erhöhtem Frakturrisiko zugelassen. Somit steht in der Europäischen Union neben Teriparatid (für Abaloparatid liegt keine Zulassung seitens der EMA vor) eine weitere osteoanabol wirksame Substanz zur Verfügung. Nach einer einjährigen Behandlung mit Romosozumab sollte eine Anschlussbehandlung mit Denosumab durchgeführt werden. Der eigentlich osteoanabole Effekt tritt jedoch nur etwa während der ersten 6 Therapiemonate auf und wird danach von einem antiresorptiven abgelöst. Die Zulassung erfolgte auf Basis von 3 klinischen Studien (FRAME, ARCH, STRUCTURE).

Die Behandlung mit Romosozumab erscheint in Bezug auf die Reduktion vertebraler und nichtvertebraler Frakturen einer alleinigen Behandlung mit Alendronat überlegen. Bei Patientinnen, die mehrjährig mit einem Antiresorptivum vorbehandelt wurden, führt eine einjährige Therapie mit Romosozumab zu einem deutlicheren Anstieg der KMD an der LWS und Hüfte als eine Behandlung mit Teriparatid. In einer der genannten Studien (ARCH) zeigte sich im Vergleich mit Alendronat ein Anstieg kardiovaskulärer Ereignisse; deswegen wurde die Indikation auf Frauen ohne Herzinfarkte und Schlaganfälle in der Anamnese eingeschränkt.

Einen vollkommen neuen Therapiezugang stellt die lokale, minimal-invasive Injektion von kalziumbasiertem, triphasischem, resorbierbaren Material in den osteoporotisch geschwächten Knochen dar. Diese als „local osteo-enhancement procedure“ (LOEP) bezeichnete (chirurgische) Intervention wird lokal jeweils einmalig durchgeführt (z. B. am Schenkelhals), hierbei wird das injizierte Material im Verlauf resorbiert und durch eigenen Knochen ersetzt [32].

## Fazit für die Praxis

- Osteoporosetherapeutika haben zur Vermeidung von zahllosen Knochenbrüchen und deren schwerwiegenden Konsequenzen geführt, können aber u. a. durch die notwendige Therapiedauer und evtl. auftretende Nebenwirkungen oder deren Fama in der Compliance/Adhärenz der Patientinnen und Patienten eingeschränkt sein.
- Neben pharmakologischen Interventionen sollte das große Feld der nichtpharmakologischen Maßnahmen nicht außer Acht gelassen werden, dass mit Fallprävention, körperlicher Aktivität zum Erhalt der Muskel- und Skelettmasse sowie Sicherungsmaßnahmen im häuslichen Bereich wesentlich dazu beitragen kann, Frakturen bei Risikopatientinnen und -patienten zu verhindern.
- In welche Richtung sich die pharmakologische Osteoporosetherapie in Zukunft entwickeln wird, hängt nicht zuletzt von laufenden neuen Ergebnissen der Grundlagenforschung ab – im Sinne einer älter werdenden Bevölkerung sind diese Forschungsansätze für viele von uns vielleicht sogar persönlich von Bedeutung.
